# HEART RATE VARIABILITY BIOFEEDBACK MODULATES PHYSIOLOGICAL INFLUENCES ON THE BOLD SIGNAL IN OLDER ADULTS

**DOI:** 10.1093/geroni/igad104.0218

**Published:** 2023-12-21

**Authors:** Richard Song, Shiyu Wang, Jungwon Min, Sarah Goodale, Ruoqi Yang, Mara Mather, Catie Chang

**Affiliations:** Vanderbilt University, Nashville, Tennessee, United States; Vanderbilt University, Vanderbilt University, Tennessee, United States; University of Southern California, Los Angeles, California, United States; Vanderbilt University, Nashville, Tennessee, United States; Vanderbilt University, Nashville, Tennessee, United States; University of Southern California, Los Angeles, California, United States; Vanderbilt University, Nashville, Tennessee, United States

## Abstract

Physiological processes, such as heart rate and breathing, impact the blood oxygen level dependent (BOLD) fMRI signal. While often regarded as noise, BOLD signal fluctuations related to physiological activity may contain valuable information about age-related brain vasculature changes including cerebrovascular arterial stiffening. This study investigates how the dynamics of BOLD responses to physiological activity change with age and in response to heart-rate variability (HRV) biofeedback training, an intervention that affects brain networks implicated in autonomic control. We used the resting-state fMRI and physiological data from the Heart Rate Variability Biofeedback Training and Emotional Regulation Dataset (n = 193). Data were split into younger (18-31 years) and older (54-80 years) age groups. Using least squares projection, we fitted physiological impulse functions to model how variation in end-tidal CO2 and heart rate propagate into BOLD signals. After permutation testing, the physiological component of BOLD signal fluctuations was significantly higher in regions including anterior cingulate cortex, basal ganglia, and white matter regions in younger adults compared to older adults. Following a 5-week HRV biofeedback training aimed to increase heart rate oscillations, areas where the physiological influence on BOLD signals increased in older adults included the prefrontal cortex and thalamus; these effects were primarily driven by greater covariance between BOLD and heart rate. In contrast, physiological effects on BOLD did not significantly change in older adults following HRV biofeedback aimed to decrease heart rate oscillations. Overall, these findings highlight how increasing HRV may improve cerebrovascular health in older adults.

